# Reply to: Relationship of circulating *Plasmodium falciparum* lifecycle stage to circulating parasitemia and total parasite biomass

**DOI:** 10.1038/s41467-022-32998-3

**Published:** 2022-09-23

**Authors:** Richard Thomson-Luque, Lasse Votborg-Novél, Nuno S. Osório, Silvia Portugal

**Affiliations:** 1 Centre of Infectious Diseases, Parasitology, Heidelberg University Hospital, Heidelberg, Germany; 2 Max Planck Institute for Infection Biology, Berlin, Germany; 3 Life and Health Sciences Research Institute (ICVS), School of Medicine, University of Minho, Portugal and ICVS/3B’s - PT Government Associate Laboratory, Braga, Portugal

**Keywords:** Parasite host response, Malaria

**replying to** M. F. Duffy et al. *Nature Communications* 10.1038/s41467-022-32996-5 (2022)

We have recently reanalysed several *P. falciparum* transcriptomic datasets with approaches centred on the tight transcriptional pattern governing *P. falciparum* along its ~48 h intraerythrocytic asexual cycle, and we showed a relation between circulation of more developed parasites within each ~48 h asexual cycle and lower parasitaemias or milder malaria symptoms^1^. Previously unpublished data from Duffy and colleagues is not fully aligned with our published conclusions. Here we discuss their comments on our recent study.

In our manuscript^1^ we included among others, the 2018 publication by Tonkin-Hill et al.^2^ which at the time did not report individual patients’ parasitaemias, total parasite biomass estimates in the form of Ptot or the levels of HRP2, haematocrit, and weight for its calculation, or that parasitaemias of the subset of the patients whose samples they used for differentially expressed gene (DEG) analysis were significantly different between samples of severe and uncomplicated malaria cases, contrary to their report for the comparison including the entire group of samples^2^, hence we could not have included these data in our publication.

Although we were aware of the different efforts Duffy and colleagues performed in Tonkin-Hill et al. to statistically normalize by developmental stage the differential expression between parasites causing severe and non-severe malaria^2^, we included their reported DEG list in the analyses of our manuscript similarly to what we did for other studies^1^. We concluded that the comparison between non-severe and severe cases without reported strong parasitaemia differences in Tonkin-Hill et al. resulted in less pronounced differences in peak of expression of DEGs and in proportions of predicted parasite stages. We agree with Duffy et al. that we could have more clearly transmitted to the reader that the normalization efforts originally applied to their dataset would prevent showing such clear trends as the other studies, and accept their criticism. Nevertheless, despite the normalizations implemented, using the reported DEG list by Tonkin-Hill et al. we still estimate higher ring-stage proportion in severe versus non-severe malaria cases (Fig. 3a, b of Ref. 1); and we observed gene expression patterns associating with number of housekeeping-gene reads, which we used instead of individual parasite levels, given that those were not originally reported (Fig. 1c of Ref. 1)^2^. Duffy et al. now report that RNAseq read counts of this housekeeping gene and individual parasitaemias do not correlate, which we had no possible way of knowing at the time of publication of Thomson-Luque et al.^1^. We do not fully agree with the additional arguments based on correlations proposed by Duffy et al. The proportion of ring- and non-ring- asexual stages is expected to sum up to 1 (or very close to 1 whenever gametocytes are present). Positive correlations to one of the groups imply negative correlations to the other, as in fact Duffy et al. observed. That the correlation found by Duffy et al. is positive with the proportion of rings, supports our rational that severe cases have higher proportion of rings (as we show in Figs. 3b and 4a of Ref. 1) and higher parasitaemias (as we show in Fig. 5a of Ref. 1). The variation of the glycine tRNA ligase shown on PlasmoDB highlights slightly lower expression of the gene at very young ring stages or schizonts, which would lead to proportionally lower normalised glycine tRNA ligase reads in parasites of younger developmental stage, and not reflect youth of parasites as proposed by Duffy and colleagues.

In Fig. 5 of Thomson-Luque et al. we have not investigated transcriptome correlates with circulating parasite density within each individual study, as we believe these would be difficult to detect due to small sample size, and short ranges in parasite densities in the vast majority of the included studies. We also did not report nor inferred any correlates between total parasite biomass and circulating parasite age in Thomson-Luque et al.^1^ as only Lee et al. reported individual values of HRP2^3^ of the ten studies included.

Duffy et al. now report that parasitaemias of the subset of the patients whose samples they analysed for differentially expressed gene (DEG) analysis were significantly different between samples of severe and uncomplicated malaria cases, however, this data is novel and different from the one they published in Tonkin-Hill et al.^2^. At the time of publishing Thomson-Luque et al. we referred to the parasite density comparison reported in Tonkin-Hill et al.^2^, showing a non-significant difference between severe and uncomplicated malaria cases *n* = 44 *p* = 0.8726^2^, and we could not foresee that the authors would now present an analysis of the same cohort with a lower sample number (*n* = 35 used on the DEG analysis) showing a significant difference (*p* = 0.0136 reported by Duffy et al.).

Regarding a possible misinterpretation of Tonkin-Hill var gene expression data, objectively, in Tonkin-Hill et al. can be read “genes involved in PfEMP1 transport and a gene involved in regulation of var genes were down-regulated in severe malaria. This suggested that var gene expression was modulated but PfEMP1 surface presentation was reduced.”^2^. And in Thomson-Luque et al. we wrote: “Tonkin-Hill et al. detected no differences between severe and uncomplicated malaria cases in total number of var gene reads, but identified segregation at the multidomain and individual domain level between severe and uncomplicated disease. However, Tonkin-Hill et al. also found genes involved in PfEMP1 transport and regulation to be downregulated in severe malaria”. We believe we did not interpret Tonkin-Hill data or statement wrongly.

We published a series of observations obtained through multiple approaches and applied to several studies, which we interpreted considering the data included in the original reports, to allow the detection of broad patterns that could otherwise be overseen. Since the publication of Thomson-Luque et al., a study investigating expression profiles of cerebral malaria and uncomplicated malaria isolates from Benin found that the mean parasite age expressed in hour post invasion was statistically lower in cerebral malaria cases, and confirmed the difference by microscopic reading of thin blood smears^4^, in line with the with the conclusions in Thomson-Luque et al. Nevertheless, we acknowledge that our combination of very different datasets may be a limitation, and we believe that future studies, including parameters like the ones now presented by Duffy and colleagues, alongside novel approaches like single-cell analysis should provide greater resolution of the parasite life-cycle stages. This may help improve interpretations and correct any unintended imperfect conclusion in Thomson-Luque et al. and the other bulk RNAseq-studies.

## Reporting summary

Further information on research design is available in the Nature Research Reporting Summary linked to this article.

## Supplementary information

Reporting Summary

## Data Availability

All data has been provided in Thomson-Luque et al.
